# Bridging the Gap of AutoGraph Between Academia and Industry: Analyzing AutoGraph Challenge at KDD Cup 2020

**DOI:** 10.3389/frai.2022.905104

**Published:** 2022-06-16

**Authors:** Zhen Xu, Lanning Wei, Huan Zhao, Rex Ying, Quanming Yao, Wei-Wei Tu, Isabelle Guyon

**Affiliations:** ^1^4Paradigm, Beijing, China; ^2^Institute of Computing Technology, Chinese Academy of Sciences, Beijing, China; ^3^Department of Computer Science, Stanford University, Stanford, CA, United States; ^4^Department of Electronic Engineering, Tsinghua University, Beijing, China; ^5^ChaLearn, Stanford, CA, United States; ^6^Laboratoire Interdisciplinaire des Sciences du Numérique (LISN), Institut National de Recherche en Informatique et en Automatique (INRIA), Centre National de la Recherche Scientifique (CNRS), University Paris-Saclay, Gif-sur-Yvette, France

**Keywords:** Graph Neural Networks, Automated Machine Learning, data challenge, node classification, graph machine learning

## Abstract

Graph structured data is ubiquitous in daily life and scientific areas and has attracted increasing attention. Graph Neural Networks (GNNs) have been proved to be effective in modeling graph structured data and many variants of GNN architectures have been proposed. However, much human effort is often needed to tune the architecture depending on different datasets. Researchers naturally adopt Automated Machine Learning on Graph Learning, aiming to reduce human effort and achieve generally top-performing GNNs, but their methods focus more on the architecture search. To understand GNN practitioners' automated solutions, we organized AutoGraph Challenge at KDD Cup 2020, emphasizing automated graph neural networks for node classification. We received top solutions, especially from industrial technology companies like Meituan, Alibaba, and Twitter, which are already open sourced on GitHub. After detailed comparisons with solutions from academia, we quantify the gaps between academia and industry on modeling scope, effectiveness, and efficiency, and show that (1) academic AutoML for Graph solutions focus on GNN architecture search while industrial solutions, especially the winning ones in the KDD Cup, tend to obtain an overall solution (2) with only neural architecture search, academic solutions achieve on average 97.3% accuracy of industrial solutions (3) academic solutions are cheap to obtain with several GPU hours while industrial solutions take a few months' labors. Academic solutions also contain much fewer parameters.

## 1. Introduction

Graph structured data has been prominent in our lives and various tasks are studied based upon, including a recommendation on Social Networks (Fan et al., 2019), traffic forecasting on road networks (Li et al., 2018), drug discovery on molecule graphs (Torng and Altman, 2019), and link prediction on the knowledge graph (Zhang et al., 2020). Graph Neural Networks (GNN) (Kipf and Welling, 2017) have been proved to be effective in modeling graph data and tremendous GNN architectures are proposed every year (Hamilton et al., 2017; Veličković et al., 2018; Wu et al., 2019; Xu et al., 2019).

When applying GNN on graph structured data, expertise and domain knowledge are often required and numerous human effort is needed to adapt to new datasets. Automated Machine Learning (AutoML) (Yao et al., 2018; Hutter et al., 2019) aims to reduce human efforts in deploying various applications. AutoML, especially Neural Architecture Search (NAS), has been successfully explored in tremendous applications, including Image Classification (Tan and Le, 2019), Object Detection (Tan et al., 2020), Semantic Segmentation (Nekrasov et al., 2019), Language Modeling (Jiang et al., 2019), and Time Series Forecasting (Chen et al., 2021). As a result, researchers have started to explore Automated Graph Neural Networks (AutoGraph). AutoGraph researchers focus mainly on automatically designing GNN architectures by NAS. The majority of these methods focus on designing the aggregation functions/layers in GNNs with different search algorithms (Zhou et al., 2019; Gao et al., 2020; Peng et al., 2020; Yoon et al., 2020; Li et al., 2021). Other works, SANE (Zhao et al., 2021) and AutoGraph (Li and King, 2020), provide the extra dimension of layer-wise skip connections design; GNAS (Cai et al., 2021), DeepGNAS (Feng et al., 2021), and Policy-GNN (Lai et al., 2020) learn to design the depth of GNNs. DiffMG (Ding et al., 2021) proposed to use NAS to search data-specific meta-graphs in the heterogeneous graph, and PAS (Wei et al., 2021) is proposed to search data-specific pooling architectures for graph classification. The recently proposed F^2^GNN (Wei et al., 2022) method decouples the design of aggregation operations with architecture topology, which is not considered before.

Despite the rich literature from academia, we ask the question of how AutoGraph is used by industrial practitioners. Toward this end, we organized the first AutoGraph challenge at KDD Cup 2020 and collaborated with 4Paradigm, ChaLearn, and Stanford University. This challenge asks participants to provide AutoGraph solutions for the node classification task. The code is executed by the platform on various graph datasets without any human intervention. Through the AutoGraph challenge, we wish to push forward the limit of AutoGraph as well as to understand the gap between industrial solutions and academic ones. In this article, we first introduce the AutoGraph challenge setting. Then, we present the winning solutions which are open sourced for everyone to use. Finally, we experiment further and compare with NAS for GNN methods and quantify empirically the gap with respect to top solutions. **We conclude three gaps of AutoGraph between academia and industry: Modeling scope, Effectiveness, and Efficiency**.

## 2. Challenge Background

### 2.1. General Statistics

The AutoGraph challenge lasted for 2 months. We received over 2200 submissions and more than 140 teams from both high-tech companies (Ant Financial, Bytedance, Criteo, Meituan Dianping, Twitter, NTT DOCOMO, etc.) and universities (MIT, UCLA, Tsinghua University, Peking University, Nanyang Technological University, National University of Singapore, IIT Kanpur, George Washington University, etc.), coming from various countries. The top three teams are aister, PASA_NJU, and qqerret. The top 10 winners' information is shown in Table 1. The 1st winner aister comes from Meituan Dianping, a company on location-based shopping and retailing service. This makes the challenge particularly valuable since we can compare academic solutions with industrial best AutoGraph practices.

**Table 1:** General information about winning teams.

**Place**	**Team name**	**Institute**		**Place**	**Team name**	**Institute**
1st	aister	Meituan Dianping		6th	SmartMN-THU	Tsinghua University
2nd	PASA_NJU	Nanjing University		6th	JunweiSun	Beijing University of Posts and Telecommunications
3rd	qqerret	Ant Financial		8th	u1234x1234	Self-employed
4th	common	Alibaba Inc.		9th	AML	Ant Financial
5th	PostDawn	Zhejiang University		10th	supergx	Nanyang Tech. University
				10th	daydayup	Hikvision Inc.

*Two teams tie in the 6th and 10th place. We list them both*.

### 2.2. Problem Formulation

The task of the AutoGraph challenge is node classification under the transductive setting. Formally speaking, consider a graph G=(V,E), where $V=\left\{v_{1},\cdots \;,v_{N}\right\}$ is the set of nodes, i.e., |V|=N and E is the set of edges, which is usually encoded by an adjacency matrix *A*∈[0, 1]^*N*×*N*^. *A*_*ij*_ is positive if there is an edge connecting from node *v*_*i*_ to node *v*_*j*_. Additionally, a matrix *X*∈ℝ^*N*×*D*^ represents the features of each node. Each node *v*_*i*_ has a class label $y_{i}\in L=\left\{1,\cdots \;,c\right\}$, resulting in the label vector $Y\in L^{N}$. In the transductive semi-supervised node classification task, part of the labels are available during training and the goal is to learn a mapping F:V→L and predict classes of unlabeled nodes.

### 2.3. Protocol

The protocol of the AutoGraph challenge is straightforward. Participants should submit a python file containing a Model class with the required fit and predict methods. We prepare an ingestion program reading the dataset and instantiate the class and call fit and predict method until the prediction finishes or the running time has reached the budget limit. The ingestion program outputs the model's prediction on test data and saves it to shared space. Then, another scoring program reads the prediction and ground truth and outputs evaluation scores. The execution of the program is always on the challenge platform. When developing locally, we provide a script to call model.py file methods directly.

### 2.4. Metric

We use Accuracy (Acc) and Balanced Accuracy (BalAcc) as evaluation metrics, defined as


$$Acc=\frac{1}{\left|\Omega \right|}\sum_{i\in \Omega }1_{{\hat{y}}_{i}=y_{i}},\;BalAcc=\frac{1}{\left|C\right|}\sum_{i\in C}Recall_{i},$$

where Ω is the set of test nodes indexes, *y*_*i*_ is the ground truth label for node *v*_*i*_, ŷ_*i*_ is the predicted label for node *v*_*i*_, *C* is the set of classes, and Recall_*i*_ is the recall score for class *i*. Accuracy (Acc) is used in the challenge to rank participants and Balanced Accuracy (BalAcc) is applied for additional analyses since it takes into account the imbalanced label distribution of datasets.

### 2.5. Datasets

A total of 15 graph datasets were used during the competition: Five public datasets were directly downloadable by the participants so they could develop their solutions offline. Five feedback datasets were made available on the platform during the feedback phase to evaluate AutoGraph algorithms on the public leaderboard. Finally, the AutoGraph algorithms were evaluated with 5 private datasets, without human intervention. These datasets are quite diverse in domains, shapes, density, and other graph properties because we expect AutoGraph solutions to have good generalization ability. On the other hand, we intentionally keep the characteristics of 5 feedback datasets and 5 private datasets similar to enable transferability. We summarize dataset statistics in Table 2. The licenses and original sources of these datasets are also provided^1^.

**Table 2:** Statistics of all datasets.

**Dataset**	**Phase**	**Domain**	**#Node**	**#Edge**	**#Feature**	**#Class**	**Avg Deg**	**Directed?**	**Weighted?**	**Skewness**
a	Public	Citation	2.7K	5.3K	1.4K	7	1.9	F	F	5
b	Public	Citation	3.3K	4.6K	3.7K	6	1.4	F	F	3
c	Public	Social	10K	733K	0.6K	41	73.3	F	F	81
d	Public	News	10K	2,917K	0.3K	20	291.7	T	T	467
e	Public	Finance	7.5K	7.8K	0	3	1.0	F	F	111
f	Feedback	Sales	10K	194K	0.7K	10	19.4	F	F	18
g	Feedback	Citation	10K	41K	8K	5	4.1	F	F	6
h	Feedback	Medicine	10K	2,461K	0.3K	23	246.1	T	T	1,773
i	Feedback	Finance	15K	16K	0	3	1.1	F	F	213
j	Feedback	Medicine	11K	22K	0	9	2.0	F	F	227
k	Private	Sales	8K	119K	0.7K	8	14.9	F	F	6
l	Private	Citation	10K	40K	7K	15	4	F	F	34
m	Private	News	10K	1,425K	0.3K	8	142.5	T	T	360
n	Private	Finance	14K	22K	0	10	1.6	F	F	61
o	Private	Social	12K	19K	0	19	1.6	F	F	62

“*Avg Deg” is the average number of edges per node. “Directed” and “Weighted” indicate the two properties of a graph. “Skewness” here is calculated by the number of nodes in the largest class divided by the number of nodes in the smallest class*.

## 3. Solutions

In this part, we introduce various methods suitable for the AutoGraph challenge, including the provided challenge baseline and solutions from top-3 winners.

### 3.1. Baseline (GCN(L2))

In the provided baseline, there is no feature engineering except for using the raw node features. For graphs without node features (e.g., dataset i and j), one hot encoding is used to unroll the node lists to a dummy feature table. During model training, an MLP is first used to map node features to the same embedding dimension. Then a two layer vanilla GCN is applied for learning node embeddings. Another MLP with softmax outputs the final classification. Dropout is used. All the hyperparameters are fixed by our experience. There is no time management since the model is simple and one full training will not cost more time than the allowed budget.

### 3.2. First Placed Winner

The 1st winner is from team aister. Their code is open source here^2^. The authors use four GNN models, two spatial ones: GraphSage (Hamilton et al., 2017) and GAT (Veličković et al., 2018), and two spectral ones: GCN (Kipf and Welling, 2017) and TAGConv (Du et al., 2017) to process node features collectively. For each GNN model, a heavy search is applied offline to determine the important hyperparameters as well as the boundaries. In the online stage, they use a smaller search space to determine the hyperparameters. In order to accelerate the search, they do not fully train each configuration but instead early stop at 16 epochs if the validation loss is not satisfactory. Additional features are used: node degrees, distribution of 1-hop and 2-hop neighbor nodes' features, etc.

### 3.3. Second Place Winner

The 2nd winner is from team PASA_NJU. Their code is open source here^3^. They also split the solution into two stages: offline stage and online stage. In the offline stage, the authors train a decision tree based on public data and other self collected datasets to classify graph types into one of three classes. Then they use GraphNAS (Gao et al., 2020) to search massively optimal GNN architectures including aggregation function, activation, number of heads in attention, and hidden units. In the online stage, the authors rapidly classify the dataset and fine tune the offline searched model.

### 3.4. Third Place Winner

The 3rd winner is from team qqerret. Their code is open source here^4^. The core model is a variant of spatial based GNN, which aggregates 2-hop neighbors of a node with additional linear parts for the node itself. Basically, the new embedding of node *i* is $ĥ\left(i\right)=\underset{j\in N_{2}\left(i\right)}{\sum }a_{j}h\left(j\right)+\alpha \left(wh\left(i\right)+b\right)$. Additionally, in the GNN output layer, a few features per node are concatenated for the final fully connected layer, including the number of edges, whether this node connects to a central node that has a lot of edges, the label distribution of 1-hop neighbor nodes, and the label distribution of 2-hop neighboring nodes.

## 4. Results

We conduct additional experiments after the AutoGraph challenge to further analyze the results. We first reproduce winning solutions and then we compare them with academic solutions. Three gaps are concluded. The first gap is presented as follows and two other gaps are concluded in section 4.2.

**Figure 1:**
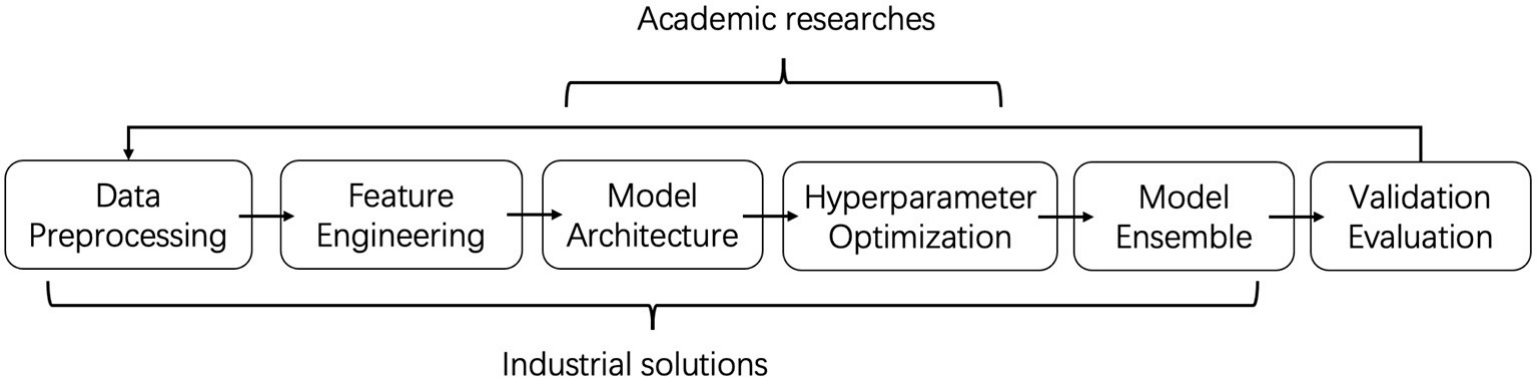
Illustration of AutoGraph scope. Industrial people provide a full pipeline solution that covers data preprocessing to evaluation. Academic researchers focus mainly on model architecture and hyperparameter optimization.

**Gap #1: Modeling Scope Is the First Gap of AutoGraph Between Academia and Industry**. In academia, researchers focus mainly on Neural Architecture Search methods to find better GNN architectures. Their contributions differ in their search space, search strategy, and evaluation methods. However, industrial solutions, e.g., 1st solution, focus more on feature engineering and model ensemble. For GNN architectures, they prefer the existing ones with little modification. In other words, industrial people provide a full pipeline solution including data preprocessing, feature engineering, model architecture, hyperparameter optimization, and model ensemble, while academic researchers focus on the model architecture part only. The gap is also illustrated in Figure 1. It might be an interesting direction for both groups to merge, i.e., AutoGraph researchers could explore the automated feature engineering and automated ensemble, and AutoGraph practitioners could adopt NAS methods for GNN.

### 4.1. Reproducing Winning Solutions

We reproduce all winning methods on all the datasets and include their results in Table 3. We observe that all three winning solutions are close in performance and all significantly beat the GCN baseline. On the other hand, in the AutoGraph challenge, due to the nature of the competition, we rank methods based on their accuracy. We state that this is not sufficient to evaluate solutions comprehensively from the scientific perspective. We add the balanced accuracy here just to show that for some methods that show close performance in accuracy, they could diverge a lot in balanced accuracy. Regarding both accuracy and balanced accuracy, we conclude that 1st solution, which comes from Meituan Dianping Company, is indeed the best among the top winners. Thus, we will later use their solutions for comparison with academic solutions. These winning solutions are already open sourced, which are reproducible and lower the barriers to using AutoGraph.

**Table 3:** Accuracy and Balanced accuracy of top methods on all datasets (%).

**Dataset**	**Phase**	**Baseline (GCN(L2))**		**1st solution**		**2nd solution**		**3rd solution**	
		**Acc**	**BalAcc**	**Acc**	**BalAcc**	**Acc**	**BalAcc**	**Acc**	**BalAcc**
a	Public	85.7	84.9	**88.5**	**87.8**	88.2	87.2	87.2	85.5
b	Public	71.4	67.8	75.2	**71.2**	**75.8**	**71.2**	75.6	69.0
c	Public	86.5	72.0	94.3	87.5	94.2	90.9	**95.4**	**91.3**
d	Public	93.7	6.1	**96.5**	**48.7**	95.1	28.8	94.6	21.0
e	Public	59.6	38.8	88.7	**92.8**	88.5	90.7	**88.8**	**92.8**
f	Feedback	86.6	78.2	**92.8**	**92.1**	92.3	**92.1**	92.4	91.4
g	Feedback	94.7	92.8	95.3	93.5	95.6	93.8	**95.8**	**94.2**
h	Feedback	90.4	8.8	**93.5**	**26.3**	92.2	17.6	92.1	16.6
i	Feedback	88.2	59.2	88.4	87.5	88.4	**92.6**	**88.5**	91.1
j	Feedback	90.7	68.1	95.9	89.0	96.1	**93.7**	**96.6**	93.3
k	Private	93.5	92.2	**95.5**	**94.4**	**95.5**	**94.4**	94.8	93.1
l	Private	90.9	84.5	**94.9**	**92.6**	94.7	91.8	94.5	**92.6**
m	Private	85.5	24.5	**98.1**	**79.7**	95.7	69.0	98.0	79.4
n	Private	85.6	47.3	**99.0**	97.3	**99.0**	**98.4**	98.9	97.0
o	Private	49.6	15.6	91.0	84.6	91.3	**90.6**	**91.4**	88.5

*The baseline is a two layer GCN. Bold values are best in comparison with other methods*.

### 4.2. Neural Architecture Search for GNN

We further adopt NAS methods for GNN and compare with the baseline and 1st solution coming from the industry. We choose the recent F^2^GNN (Wei et al., 2022) in our experiment, which searches for data-specific GNN topology. To compare fairly with GCN baselines, we fix the aggregation to GCN and search only the GNN topology, which we call F^2^GCN. Since F^2^GCN requires at least 4 layers, we also run a 4 layer GCN baseline for better comparison. The results are given in Table 4.

**Table 4:** Accuracy comparison of GCN baselines, F^2^GCN, and industrial best solution (%).

**Dataset**	**GCN(L2)**	**GCN(L4)**	**F^2^GCN(L4)**	**1st solution**
a	85.7	84.4	84.4 (95.4)	88.5 (100)
b	71.4	70.5	71.3 (94.8)	75.2 (100)
c	86.5	82.3	92.8 (98.4)	94.3 (100)
d	93.7	93.6	93.9 (97.3)	96.5 (100)
e	59.6	87.5	88.4 (99.7)	88.7 (100)
f	86.6	87.6	92.1 (99.2)	92.8 (100)
g	94.7	93.4	95.3 (100)	95.3 (100)
h	90.4	90.3	90.1 (96.4)	93.5 (100)
i	88.2	87.6	88.3 (99.9)	88.4 (100)
j	90.7	83.6	95.3 (99.4)	95.9 (100)
k	93.5	93.2	93.4 (97.9)	95.5 (100)
l	90.9	89.1	92.9 (97.9)	94.9 (100)
m	85.5	86.1	86.1 (87.8)	98.1 (100)
n	85.6	95.2	96.7 (97.7)	99.0 (100)
o	49.6	71.8	88.8 (97.6)	91.0 (100)
Avg			− (97.3)	− (100)

*L2, L4 mean 2, and 4 layers for the GNN architecture. Numbers in parentheses are the relative accuracy with respect to 1st solution. We regard 1st solution as 100%. The last line is the average percentage*.

**Figure 2:**
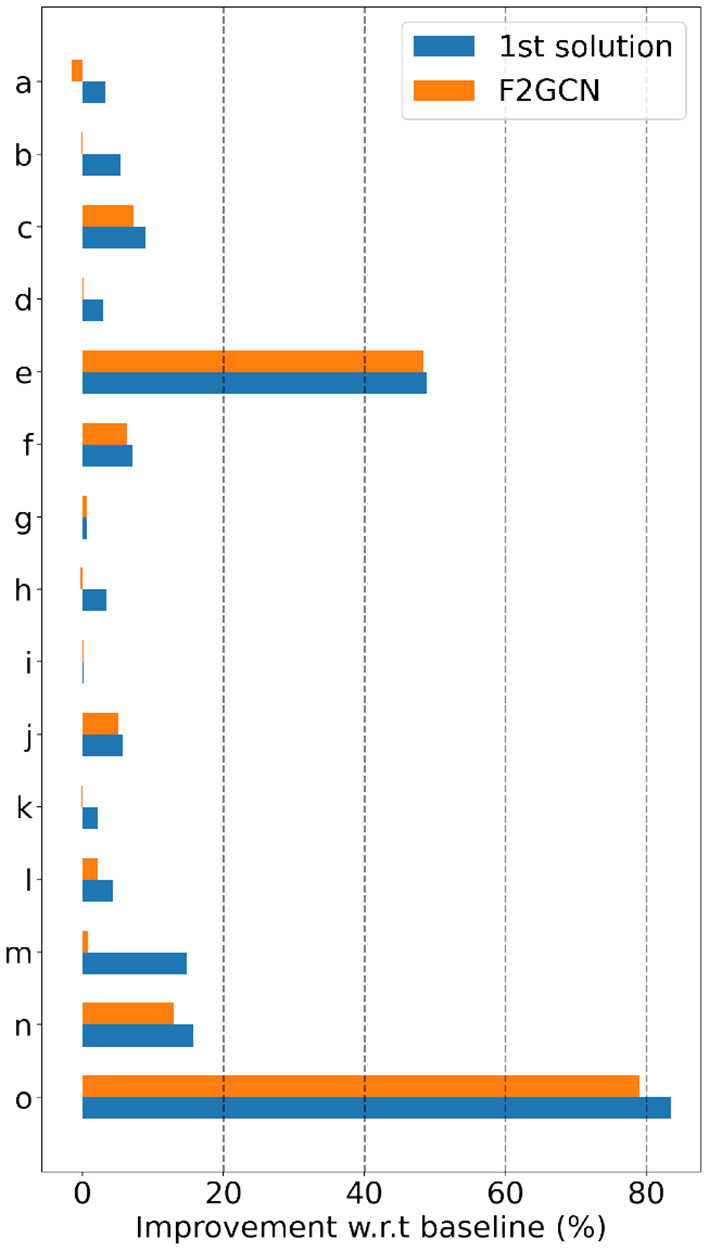
Accuracy improvement with respect to baseline.

**Gap #2: Effectiveness Is the Second Gap of AutoGraph Between Academia and Industry**. We observe from Figure 2 and Table 4 that all baselines and F^2^GCN methods are not as good as 1st winning solution. However, for many datasets, e.g., e, f, g, i, j, F^2^GCN is very close to the best industrial solution. On average, F^2^GCN which focuses only on architecture search reaches 97.3% of the best solution. Note that the 1st solution constructs additional node features and uses multiple GNN architectures for ensemble while F^2^GCN does not use any feature engineering or model ensemble. This shows the effectiveness of the winner's engineering practices and F^2^GCN's adaptive architecture search. The winning teams also have access to public datasets and the public leaderboard to iteratively fine tune their methods. F^2^GCN does not assume any prior knowledge of the datasets, which shows further its effectiveness.

To better understand the solutions, we calculate the number of parameters of the baseline, F^2^GCN, and the 1st solution, as shown in Table 5.

**Table 5:** Number of parameters of baseline, 1st solution and F^2^GCN (Unit: Millions).

**Dataset**	**GCN(L2)**	**F^2^GCN(L4)**	**1st solution**
a	0.023	0.908 (75.7)	1.199 (100)
b	0.059	0.700 (44.2)	1.583 (100)
c	0.011	1.598 (98.0)	1.631 (100)
d	0.006	0.042 (3.20)	1.296 (100)
e	0.121	0.354 (31.8)	1.114 (100)
f	0.013	0.039 (2.30)	1.688 (100)
g	0.134	0.313 (13.1)	2.389 (100)
h	0.006	0.271 (20.9)	1.294 (100)
i	0.241	2.269 (113.0)	2.013 (100)
j	0.171	0.834 (60.6)	1.376 (100)
k	0.012	1.478 (108.0)	1.395 (100)
l	0.108	0.614 (25.6)	2.395 (100)
m	0.005	0.010 (0.80)	1.278 (100)
n	0.218	0.488 (27.8)	1.756 (100)
o	0.192	0.822 (52.5)	1.565 (100)
Avg		− (45.1)	− (100)

*Numbers in parentheses are the relative number of parameters with respect to 1st solution. We regard 1st solution as 100%. The last line is the average percentage*.

**Figure 3:**
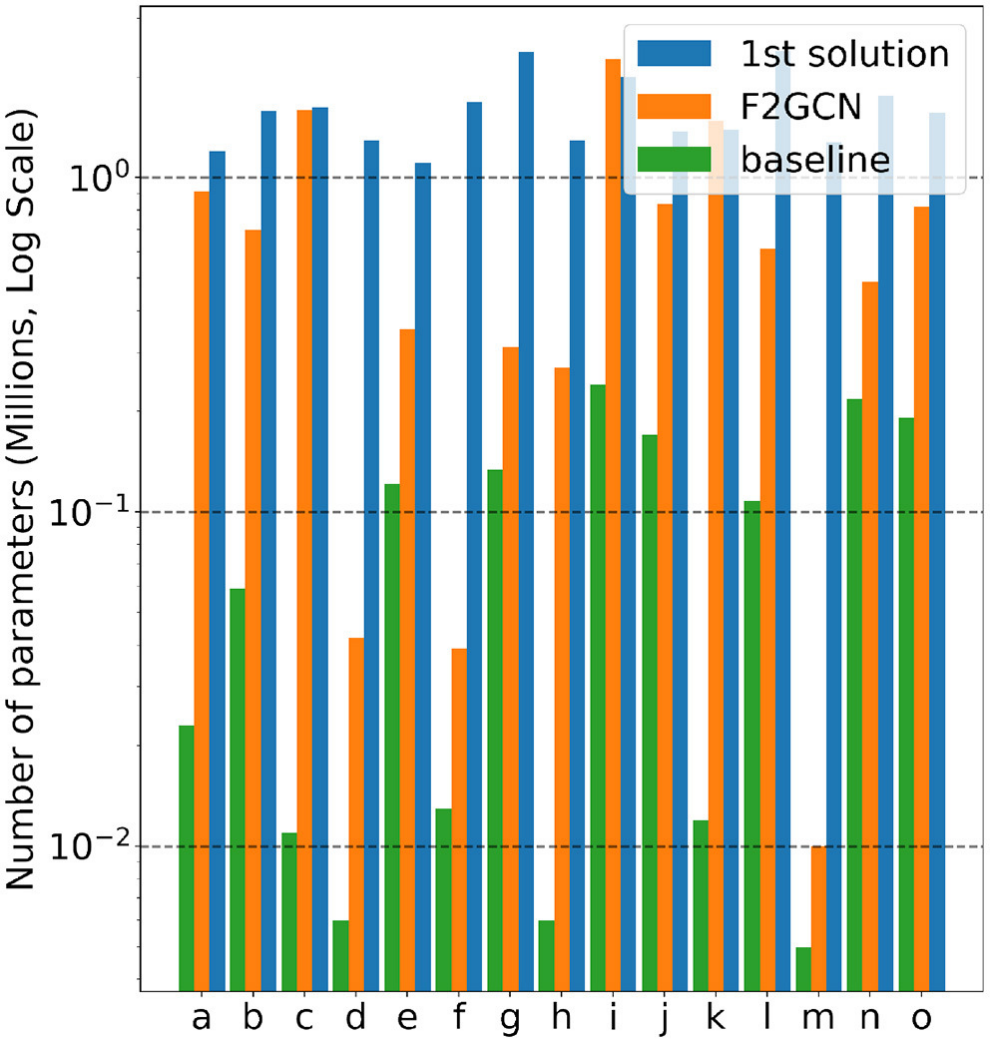
Comparison of the number of parameters of baseline, 1st solution, and F^2^GCN (log scale).

**Gap #3: Efficiency Is the Third Gap of AutoGraph Between Academia and Industry**. From Figure 3 and Table 5, F^2^GCN uses significantly fewer parameters than the best industrial solution on most datasets (13 out of 15). On average, F^2^GCN consumes 45.1% of the 1st solution in terms of parameter size, which is quite resource-efficient. Note that feature engineering and ensemble do not contain additional parameters and basically, F^2^GCN searches one GNN model to compete with the ensemble of 4 types of GNN models in the 1st solution. As for time devotion, the winning solutions come from a team's months of work, which consists of 5 or more members. F^2^GCN only runs for a few GPU hours per dataset, demonstrating its time efficiency compared to industrial solutions.

## 5. Conclusion

We organized the first Automated Graph Learning (AutoGraph) Challenge at KDD Cup 2020. We presented in this article its settings, dataset, and solutions, which are all open sourced. Furthermore, we ran additional post-challenge experiments to compare the baseline [Graph Convolution Network (GCN)], the winning solution (feature engineering-based ensemble of various Graph Neural Networks), and a recent and efficient Neural Architecture Search (NAS) for the GNN method called F^2^GCN. This article provides results that could bridge the gap between academic research and industrial practices, by correcting the bias favoring certain approaches. This gap is currently at 3 aspects: **Gap #1 modeling scope**. (academia focuses more on model-centric approaches, emphasizing NAS; industry emphasizes data centric approaches and feature engineering); **Gap #2 effectiveness**. (academic solutions are perceived by the industry to be less effective than their own counterpart); **Gap #3 efficiency**. (academic solutions are perceived to be parsimonious or slower than industrial solutions). Our results indicate that the “academic” NAS-based approach that we applied attains performances closely matching those of the winning industrial solution while being both faster and more parsimonious in the number of parameters, therefore, closing Gap #2 and #3. Moreover, we hope that these results will help reduce Gap #1, by encouraging industrial practitioners to apply NAS methods (and particularly F^2^GCN), eventually combining the best of both approaches. We believe the results we obtained are significant since they involve a benchmark on 15 datasets.

## Data Availability Statement

The datasets presented in this study can be found in online repositories. The names of the repository/repositories and accession number(s) can be found below: https://github.com/AutoML-Research/AutoGraph-KDDCup2020.

## Author Contributions

ZX, LW, and HZ contributed to post-challenge experiments and the first draft of the manuscript. RY, WWT, and IG contributed to further discussions. All authors contributed to the application, organization of the AutoGraph challenge at KDD Cup 2020, contributed to manuscript revision, read, and approved the submitted version.

## Funding

Funding and support have been received by several research grants, including 4Paradigm, Big Data Chair of Excellence FDS Paris-Saclay, Paris Région Ile-de-France, and ANR Chair of Artificial Intelligence HUMANIA ANR-19-CHIA-0022, ChaLearn, Microsoft, Google. Microsoft and Google provide cloud computing resources for hosting the challenge and post-challenge experiments.

## Conflict of Interest

ZX, LW, HZ, and WWT are employed by 4Paradigm, China. The funder 4Paradigm had the following involvement in the study: prepared the challenge, post-challenge analysis and paper writing. The remaining authors declare that the research was conducted in the absence of any commercial or financial relationships that could be construed as a potential conflict of interest.

## Publisher's Note

All claims expressed in this article are solely those of the authors and do not necessarily represent those of their affiliated organizations, or those of the publisher, the editors and the reviewers. Any product that may be evaluated in this article, or claim that may be made by its manufacturer, is not guaranteed or endorsed by the publisher.

## Footnotes

^1^ https://github.com/AutoML-Research/AutoGraph-KDDCup2020

^2^ https://github.com/aister2020/KDDCUP_2020_AutoGraph_1st_Place

^3^ https://github.com/Unkrible/AutoGraph2020

^4^ https://github.com/white-bird/kdd2020_GCN

## References

[B1] Cai S. Li L. Deng J. Zhang B. Zha Z.-J. Su L.. (2021). “Rethinking graph neural network search from message-passing,” in CVPR 2021.

[B2] Chen D. Chen L. Shang Z. Zhang Y. Wen B. Yang C. (2021). ‘Scale-aware neural architecture search for multivariate time series forecasting,” in CoRR 2021. Available online at: https://arxiv.org/abs/2112.07459

[B3] Ding Y. Yao Q. Zhao H. Zhang T. (2021). “Diffmg: differentiable meta graph search for heterogeneous graph neural networks,” in KDD 2021.

[B4] Du J. Zhang S. Wu G. Moura J. M. F. Kar S. (2017). “Topology adaptive graph convolutional networks,” in CoRR 2017. Available online at: https://arxiv.org/abs/1710.10370

[B5] Fan W. Ma Y. Li Q. He Y. Zhao Y. E. Tang J.. (2019). “Graph neural networks for social recommendation,” in WWW 2019 (San Francisco, CA).

[B6] Feng G. Wang C. Wang H. (2021). Search for deep graph neural networks. arXiv:2109.10047. 10.48550/arXiv.2109.10047

[B7] Gao Y. Yang H. Zhang P. Zhou C. Hu Y. (2020). “Graph neural architecture search,” in IJCAI 2020 (Yokohama, Japan).

[B8] Hamilton W. Ying Z. Leskovec J. (2017). “Inductive representation learning on large graphs,” in NIPS 2017 (Long Beach, CA).

[B9] Hutter F. Kotthoff L. Vanschoren J. (2019). Automated machine learning: methods, systems, challenges. Berlin: Springer Nature.

[B10] Jiang Y. Hu C. Xiao T. Zhang C. Zhu J. (2019). “Improved differentiable architecture search for language modeling and named entity recognition,” in EMNLP-IJCNLP 2019 (Hong Kong, China).

[B11] Kipf T. N. Welling M. (2017). “Semi-supervised classification with graph convolutional networks,” in ICLR 2017 (Toulon, France).

[B12] Lai K.-H. Zha D. Zhou K. Hu X. (2020). “Policy-gnn: aggregation optimization for graph neural networks,” in KDD 2020.

[B13] Li Y. King I. (2020). “Autograph: automated graph neural network,” in ICONIP 2020 (Bangkok, Thailand).

[B14] Li Y. Wen Z. Wang Y. Xu C. (2021). “One-shot graph ne‘ural architecture search with dynamic search space,” in AAAI 2021.

[B15] Li Y. Yu R. Shahabi C. Liu Y. (2018). “Diffusion convolutional recurrent neural network: data-driven traffic forecasting,” in ICLR 2018 (Vancouver, Canada).

[B16] Nekrasov V. Chen H. Shen C. Reid I. D. (2019). “Fast neural architecture search of compact semantic segmentation models via auxiliary cells,” in CVPR 2019 (Long Beach, CA).

[B17] Peng W. Hong X. Chen H. Zhao G. (2020). “Learning graph convolutional network for skeleton-based human action recognition by neural searching,” in AAAI 2020 (New York, NY).

[B18] Tan M. Le Q. V. (2019). “Efficientnet: rethinking model scaling for convolutional neural networks,” in ICML 2019 (Long Beach, CA).

[B19] Tan M. Pang R. Le Q. V. (2020). “Efficientdet: scalable and efficient object detection,” in CVPR 2020.

[B20] Torng W. Altman R. B. (2019). Graph convolutional neural networks for predicting drug-target interactions. J. Chem. Inf. Model. 59, 4131–4149. 10.1021/acs.jcim.9b00628 31580672

[B21] Veličković P. Cucurull G. Casanova A. Romero A. Liò P. Bengio Y. (2018). “Graph attention networks,” in ICLR 2018 (Vancouver, Canada).

[B22] Wei L. Zhao H. He Z. (2022). “Designing the topology of graph neural networks: a novel feature fusion perspective,” in WWW 2022.

[B23] Wei L. Zhao H. Yao Q. He Z. (2021). “Pooling architecture search for graph classification,” in CIKM 2021.

[B24] Wu F. Souza A. Zhang T. Fifty C. Yu T. Weinberger K. (2019). “Simplifying graph convolutional networks,” in ICML 2019 (Long Beach, CA).

[B25] Xu K. Hu W. Leskovec J. Jegelka S. (2019). “How powerful are graph neural networks?” in ICLR 2019 (New Orleans, LA).

[B26] Yao Q. Wang M. Escalante H. J. Guyon I. Hu Y. Li Y.. (2018). Taking human out of learning applications: a survey on automated machine learning. arXiv:1810.13306. 10.48550/arXiv.1810.13306

[B27] Yoon M. Gervet T. Hooi B. Faloutsos C. (2020). “Autonomous graph mining algorithm search with best speed/accuracy trade-off,” in ICDM 2020 (Sorrento, Italy).

[B28] Zhang Z. Zhuang F. Zhu H. Shi Z. Xiong H. He Q. (2020). “Relational graph neural network with hierarchical attention for knowledge graph completion,” in AAAI 2020 (New York, NY).

[B29] Zhao H. Yao Q. Tu W. (2021). “Search to aggregate neighborhood for graph neural network,” in ICDE 2021.

[B30] Zhou K. Song Q. Huang X. Hu X. (2019). Auto-gnn: neural architecture search of graph neural networks. arXiv:1909.03184. 10.48550/arXiv.1909.03184 PMC9714572 36466713

